# Knowledge, attitude, and acceptance regarding COVID-19 vaccines in Sudan

**DOI:** 10.3389/fpubh.2022.954810

**Published:** 2022-10-10

**Authors:** Hazeem A. Mohmmed, Ragda Abdalmonem Alawad, Ahmed K. Awad, Abdalaziz Awad Alobied

**Affiliations:** ^1^Faculty of Medicine, Ain Shams University, Cairo, Egypt; ^2^Faculty of Medicine, Karary University, Khartoum, Sudan; ^3^Faculty of Medicine, Omdurman Islamic University, Khartoum, Sudan

**Keywords:** knowledge, attitude, acceptance, COVID-19 vaccines, Sudan

## Abstract

**Background:**

COVID-19 is a respiratory disease caused by SARS-CoV-2, a new coronavirus discovered in December 2019 in China. COVID-19 symptoms are similar to those of viral flu but may be more severe, these symptoms can be defended by vaccines, the most distributed 6 candidate vaccines are Pfizer, BioNTech, Moderna, Johnson & Johnson/Janssen AstraZeneca, Sinopharm, Sinovac. In Sudan, the virus has rapidly spread in the country, causing a total of 37,138 confirmed cases with 2,776 deaths till July 21, 2021. We are targeting health workers, medical students, and the general public to assess their behavior regarding COVID-19 vaccines in Sudan, recognize the determinants of their behavior, and identify the factors increasing vaccine acceptance among them.

**Methods:**

We conducted a pretested cross-sectional online survey involving healthcare workers, medical students, and the general population in Sudan in July and August. We collected the data by sending the survey to social media platforms (e.g., Facebook and WhatsApp). The survey was conducted anonymously without identity-related data. We used both convenience sampling and snowball sampling methods as the participants were asked to forward the survey link to their contacts. The sample size was calculated using Slovin's formula and we estimated the sample size to be 400.

**Results:**

Of our 400 participants, 36.8% (*n* = 147) were males and 63.2% (*n* = 253) were females, the mean age of the participants was 24.17 ± 8.07. The overall vaccine acceptance rate was 48.2% (*n* = 193) and “Occupation” was the only sociodemographic domain significantly associated with vaccine acceptance, showing a higher acceptance rate among health care workers (*p* = 0.009). “Afraid of unknown side effects” was the most commonly reported barrier to vaccination (*p* = 0.33).

**Conclusion:**

The vaccine acceptance rate is low, and public health authorities and the government in Sudan have a heavy mission for implementing successful vaccination programs with high coverage.

## Introduction

COVID-19 is a respiratory disease caused by SARS-CoV-2, a new coronavirus discovered in the year 2019 (1). It was first detected in China in December 2019 and declared a pandemic by the World Health Organization (WHO) on March 11, 2020 (2).

The virus spreads mainly from person to person through respiratory droplets produced when an infected person coughs, sneezes, or talks (1). It appears with a variety of symptoms including fever, cough, headache, muscle ache, loss of taste or smell, nausea, vomiting, diarrhea, etc. COVID-19 symptoms are similar to those of viral flu but may be more severe (3). All these can be defended by vaccines, as there are about 176 candidate vaccines (4). Most vaccines are in the clinical trial phase, and few have already gained Emergency Use Authorization (EUA) (5).

The most distributed 6 candidate vaccines are Pfizer, BioNTech, Moderna, Johnson & Johnson / Janssen, AstraZeneca, Sinopharm, and Sinovac, but three of those are authorized and recommended in the USA: Pfizer, BioNTech, Moderna, Johnson & Johnson / Janssen (6). With common side effects ranging from fever and nausea to tiredness, headache, muscle pain, and chills. All these vaccines are freely available in numerous primary health care centers all over Sudan.

In Sudan, the virus has rapidly spread in the country, causing a total of 62251 confirmed cases with 4941 deaths until May 26, 2022 (7).In this study, we are targeting health workers, medical students, and the general public to assess their behavior regarding the COVID-19 vaccine in Sudan. Numerous studies have shown several factors responsible for vaccine acceptance when a new vaccine is introduced (8–11). These include the safety and efficacy of the vaccine, adverse health outcomes, misconceptions about the need for vaccination, lack of trust in the health system, and lack of knowledge among the community on vaccine-preventable diseases (11, 12). Accordingly, we conducted a cross-sectional study on the Sudanese population. To our knowledge, this is the first study conducted in Sudan aiming to determine the behavior of health workers, medical students, and the general public regarding COVID-19 vaccines, recognize the determinants of their behavior, and identify the factors increasing vaccine acceptance among them. Furthermore, this study could represent a guide for healthcare organizations and public health experts in Sudan to highlight the expected challenges for COVID-19 vaccination.

## Materials and methods

### Study design

We conducted a pretested cross-sectional online survey involving healthcare workers, medical students, and the general population in Sudan in July and August. We collected the data by sending the survey to social media platforms (e.g., Facebook and WhatsApp). The survey was conducted anonymously without identity-related data, we followed the Strengthening the Reporting of Observational Studies in Epidemiology statement guidelines. We categorized the participants by occupation into health care workers, medical students, and the general population.

### Sample size and sampling technique

According to the United Nations data and World ometers, the Sudanese population was 43,849,260 as of December 31, 2020. To reach participants, we used both convenience sampling and snowball sampling methods as the participants were asked to forward the survey link to their contacts. The sample size was calculated using Slovin's formula, considering a sample proportion of 50%, wherein n = required sample size [*n* = *N*/(1+Ne∧2)] with 95% CI and 5% margin of error. Therefore, we required sample size of 400.

### Inclusion and exclusion criteria

Only Sudanese nationals currently living in Sudan, and those aged ≥16 years were included. Non-Sudanese or those who are Sudanese and currently not living in Sudan or those who live in Sudan and non-Sudanese were excluded.

### Study tools

We conducted a pilot test with 36 random participants to confirm the practicability, validity, and interpretation of the responses, the final version of the survey consisted of the following sections:

- **Informed consent section:** The first page of the first section consisted of study information and an informed consent agreement. Only the participants signing the consent were able to continue the survey.
- **Sociodemographic section:** These questions were related to gender, age, occupation, geographical residency region, suspicion, and confirmation of previous COVID-19 infection.
- **Knowledge, attitudes, and practices regarding the COVID-19 pandemic and vaccines:** This section was developed based on literature reviews of relevant studies (13–17). This section consisted of 8 questions with 3, 4, and 1 questions in the knowledge, attitude, and acceptance items, respectively, with each correct answer, assigned a score of one and a score of zero for each incorrect answer. The questionnaire was valid and reliable with a *p*-value of < 0.001 and good internal consistency with a Cronbach's alpha value of 0.819, we determined scores of 66.7% (2/3), 75% (3/4), and 100% (1/1) as a cut-off score for adequate knowledge, attitude and acceptance respectively.
- **Barriers to vaccine acceptance:** This section consisted of 6 questions to assess the possible barriers that could contribute to vaccine refusal, the barriers included fear of side effects, conspiracy theory, and current/previous COVID-19 infection.

### Statistical analysis

Collected data were revised, coded, entered on a PC, and analyzed using SPSS version 16. The collected data were analyzed according to the type of variables. Quantitative variables will be presented by their mean and standard deviation and analyzed by *t*-test. Qualitative variables will be presented by frequency and analyzed by a chi-square test. The level of significance adopted for this study is *p*-value < 0.05.

## Results

### Socio-demographic characteristics

Of our 400 participants, 36.8% (*n* = 147) were males and 63.2% (*n* = 253) were females. 91.2% (*n* = 365) of the participants were in the age group A (16–35 years old), 7.8% (*n* = 31) of the participants were in the age group B (36–55 years old), and only 1% (*n* = 4) of the participants were in the age group C (56–76 years old). The mean age of the participants was 24.17 years with a standard deviation of 8.07. Medical Students represent 51.55% (*n* = 206) of the participants, health care workers represent 14% (*n* = 56) of the participants, and 34.5% (*n* = 138) are from the general public. Moreover, 83.5% (*n* = 334) of the participants resided in the capital of Sudan (Khartoum state) and 16.5% (*n* = 66) resided in the rest of the states. Only 6% (*n* = 24) of the participants suffered from chronic diseases such as diabetes mellitus and hypertension. While 29.2% (*n* = 117) of the participants were suspected to have COVID-19, only 5.2% (*n* = 21) of them were confirmed to have the disease by RT-PCR (Table 1).

**Table 1:** Sociodemographic characteristics of the participants.

**Variable**	**Total**	**Healthcare workers**	**Medical students**	**General population**	***P*-value**
	***n* = 400 (%)**	***n* = 56 (%)**	***n* = 206 (%)**	***n* = 138 (%)**	
**Age group in years**					< 0.001*
16–35	365 (91.2%)	47 (83.9%)	206 (100%)	112 (81.1%)	
36–55	31 (7.8%)	9 (16.1%)	0 (0.0%)	22 (16.0%)	
56–76	4 (1%)	0 (0.0%)	0 (0.0%)	4 (2.9%)	
**Gender**					< 0.001*
Male	147 (36.8%)	11 (19.6%)	58 (28.2%)	78 (56.5%)	
Female	253 (63.2%)	45 (80.4%)	148 (71.8%)	60 (43.5%)	
**Residency**					0.085
Khartoum	334 (83.5%)	42 (75.0%)	179 (86.9%)	113 (81.9%)	
Other states	66 (16.5%)	14 (25.0%)	27 (13.1%)	25 (18.1%)	
**Presence of chronic disease**					0.927
Yes	24 (6.0%)	4 (7.1%)	12 (5.8%)	8 (5.8%)	
No	376 (94%)	52 (92.9%)	194 (94.2%)	130 (94.2%)	
**Suspected for COVID-19**					0.180
Yes	117 (29.2%)	22 (39.3%)	59 (28.6%)	36 (26.1%)	
No	283 (70.8%)	34 (60.7%)	147 (71.4%)	102 (73.9%)	
**Confirmed COVID-19**					0.668
Yes	21 (5.2%)	4 (7.1%)	9 (4.4%)	8 (5.8%)	
No	379 (94.8%)	52 (92.9%)	197 (95.6%)	130 (94.2%)	

*Statistically significant.

### Knowledge, attitude, and acceptance regarding COVID-19 and COVID-19 vaccines

Overall, the mean ± SD scores for knowledge, attitude, and acceptance were 1.34 ± 0.45 (ranging from 0 to 3), 1.20 ± 1.67 (ranging from 0 to 4), and 0.48 ± 0.49 (ranging from 0 to 1), respectively Table 2 summarize the distribution of knowledge, attitude, and acceptance scores per occupation.

**Table 2:** Knowledge, attitude, and acceptance scores by occupation.

**Variable**	**Total**	**HCWs**	**Medical students**	**General population**	***P*-value**
	***n* = 400 (%)**	***n* = 56 (%)**	***n* = 206 (%)**	***n* = 138 (%)**	
**Knowledge**					0.001*
0	0 (0.0%)	0 (0.0%)	0 (0.0%)	0 (0.0%)	
1	262 (65.5%)	43 (76.8%)	117 (56.8%)	102 (73.9%)	
2	138 (34.5%)	13 (23.2%)	89 (43.2%)	36 (26.1%)	
3	0 (0.0%)	0 (0.0%)	0 (0.0%)	0 (0.0%)	
Mean ± SD	1.34 ± 0.45	1.23 ± 0.42	1.43 ± 0.49	1.26 ± 0.44	
**Attitude**					< 0.001*
0	243 (60.7%)	36 (64.3%)	113 (54.9%)	94 (68.1%)	
1	27 (6.8%)	1 (1.8%)	23 (11.2%)	3 (2.2%)	
2	17 (4.2%)	6 (10.7%)	8 (3.9%)	3 (2.2%)	
3	31 (7.8%)	1 (1.8%)	30 (14.5%)	0 (0.0%)	
4	82 (20.5%)	12 (21.4%)	32 (15.5%)	38 (27.5%)	
Mean ± SD	1.20 ± 1.67	1.14 ± 1.66	1.2 4± 1.58	1.16 ± 1.78	
**Acceptance**					0.011*
0	207 (51.8%)	20 (35.7%)	105 (51.0%)	82 (59.4%)	
1	193 (48.2%)	36 (64.3%)	101 (49.0%)	56 (40.6%)	
Mean ± SD	0.48 ± 0.49	0.64 ± 0.48	0.4 9± 0.50	0.40 ± 0.49

*Statistically significant.

In response to the question “What is the origin of the new coronavirus?” Only 34.5% (*n* = 138) of the participants think that the virus is natural and 65.5% (*n* = 262), we found that 33.8% (*n* = 135) of the participants believe that the pandemic is exaggerated to benefit pharmaceutical companies and 37.2% (*n* = 149) reported that the source of their knowledge regarding COVID-19 and COVID-19 vaccines are scientific articles and organizations e.g.; WHO and CDC and 62.8% (*n* = 251) reported that the source of their knowledge regarding COVID-19 and COVID-19 vaccines are T.V shows/news or social media. Table 3 summarizes the distribution of knowledge per occupation.

**Table 3:** Knowledge, attitude, and acceptance regarding COVID-19 and COVID-19 vaccines by occupation.

**Variable**	**Total**	**Healthcare workers**	**Medical students**	**General population**	***P*-value**
	***n* = 400 (%)**	***n* = 56 (%)**	***n* = 206 (%)**	***n* = 138 (%)**	
**Knowledge**					
What is the origin of the new coronavirus?					0.001*
Natural virus	138 (34.5%)	13 (23.2%)	89 (43.2%)	36 (26.1%)	
Synthetic virus	85 (21.2%)	22 (39.3%)	26 (12.6%)	37 (26.8%)	
Synthetically developed natural virus	177 (44.2%)	21 (37.5%)	91 (44.2%)	65 (47.1%)	
**Is the pandemic exaggerated to benefit pharmaceutical companies?**					0.015*
Yes	135 (33.8%)	26 (46.4%)	57 (27.7%)	52 (37.7%)	
No	265 (66.2%)	30 (53.6%)	149 (72.3%)	86 (62.3%)	
**What is the source of your knowledge regarding COVID-19/vaccines?**					0.041*
Scientific articles/ organizations	149 (37.2%)	28 (50.0%)	80 (38.8%)	41 (29.7%)	
T.V shows and news	135 (33.8%)	13 (23.2%)	74 (35.9%)	48 (34.8%)	
Social media	116 (29.0%)	15 (26.8%)	52 (25.3%)	49 (35.5%)	
Attitude					
**COVID-19 vaccine should be mandatory for the whole population**					0.024*
Yes	83 (20.8%)	13 (23.2%)	32 (15.5%)	38 (27.5%)	
No	317 (79.2%)	43 (76.8%)	174 (84.5%)	100 (72.5%)	
**COVID-19 vaccine should be Optional for the whole population**					0.084
Yes	125 (31.2%)	19 (33.9%)	16 (30.1%)	44 (31.9%)	
No	275 (68.8%)	37 (66.1%)	190 (69.9%)	94 (68.1%)	
**COVID-19 vaccine should be mandatory for HCWs**					0.014*
Yes	154 (38.5%)	20 (35.7%)	93 (45.1%)	41 (29.7%)	
No	246 (61.5%)	36 (64.3%)	113 (54.9%)	97 (70.3%)
**COVID-19 vaccine should be Mandatory for old aged**					0.141
Yes	120 (30.0%)	12 (21.4%)	70 (34.0%)	38 (27.5%)	
No	280 (70.0%)	44 (68.6%)	136 (66.0%)	100 (72.5%)	
**Acceptance**					
**Did you take/ intends to take the COVID-19 vaccine?**					0.011*
Yes, I took the vaccine	54 (13.5%)	21 (37.5%)	25 (12.1%)	8 (5.8%)	
Yes, I intend to take the vaccine	139 (34.75%)	15 (26.8%)	76 (36.9%)	48 (34.8%)	
No	207 (51.75%)	20 (35.7%)	105 (51.0%)	82 (59.4%)	

*Statistically significant.

20.8% (*n* = 83) of the participants reported that COVID-19 vaccine should be mandatory for the whole population, 38.5% (*n* = 154) reported that should be mandatory for health care workers, and 30% (*n* = 120) reported that should be mandatory for old aged and 31.2% (*n* = 125) reported that COVID-19 vaccines should be optional for the whole population. Table 3 summarizes the distribution of attitudes per occupation.

The overall vaccine acceptance rate was 48.2% (*n* = 193). We used socio-demographics to analyze variation in vaccine acceptance, thus we tested the socio-demographic determinants by chi-square test and multivariable binomial logistic regression, except for occupation (*P* = 0.011 by chi-square and 0.009 by multivariable binomial logistic regression). We found no other statistically significant associations (Table 4).

**Table 4:** Sociodemographic determinants of vaccine acceptance.

**Variable**	**Vaccine Acceptance *n* (%)**	**A *p*-value of the chi-square test**	**Multivariable OR (CI)**	**A *p*-value of multivariable binary logistic regression**
**Age in years (** ***n*** **)**		0.167		0.107
16–35 (365)	171 (46.8%)		1.0 (Reference)	
36–55 (31)	20 (64.5%)		0.410 (0.178–0.947)	
56–76 (3)	2 (50.0%)		0.596 (0.079–4.477)	
**Gender**		0.847		0.898
Male (147)	70 (47.6%)		1.0 (Reference)	
Female (253)	123 (48.6%)		1.030 (0.656–1.616)	
**Occupation**		0.011*		0.009*
Health care workers (56)	36 (64.3%)		1.0 (Reference)	
Medical students (206)	101 (49%)		1.628 (0.866–3.060)	
The general population (138)	56 (40.6%)		2.743 (1.395–5.394)	
**Residency**		0.561		0.582
Khartoum (334)	159 (47.6%)		1.0 (Reference)	
Other states (66)	34 (51.5%)		0.856 (0.492–1.488)	
**Chronic diseases**		0.860		0.971
Yes (24)	12 (50.0%)		1.0 (Reference)	
No (376)	181 (48.1%)		1,016 (0.437–2.395)	

*Statistically significant.

### Barriers to COVID-19 vaccine acceptance

Out of the participants, 48% (*n* = 192) reported that they are afraid of unknown side effects of the vaccines, 4.2% (*n* = 17) reported that they have a good immunity, 4.8% (*n* = 19) believed that the pandemic/vaccines are a conspiracy, 5% (*n* = 20) believes that vaccines contain a brain control chip, 3% (*n* = 12) reported that they have/had COVID-19, and 3% (*n* = 12) reported their chronic disease as barrier for vaccination. We used occupation to analyze barriers to vaccine acceptance using a chi-square test, we summarized the findings in Table 5.

**Table 5:** Barriers to vaccine acceptance by population characteristics.

**Variable**	**Total**	**Healthcare workers**	**Medical students**	**General population**	***P*-value**
	***n* = 400 (%)**	***n* = 56 (%)**	***n* = 206 (%)**	***n* = 138 (%)**	
Afraid of unknown side effects	192 (48%)	18 (32.1%)	105 (51.0%)	69 (50.0%)	0.033*
I have a good immunity	17 (4.25%)	3 (5.4%)	9 (4.4%)	5 (3.6%)	0.857
Pandemic/vaccine is a conspiracy	19 (4.75%)	3 (5.4%)	8 (3.9%)	8 (5.8%)	0.697
The vaccine contains a brain control chip	20 (5.0%)	0 (0.0%)	8 (3.9%)	12 (8.7%)	0.024*
I have/had COVID-19	12 (3.0%)	1 (1.8%)	7 (3.4%)	4 (2.9%)	0.536
I have a chronic disease	12 (3.0%)	2 (3.6%)	5 (2.4%)	5 (3.6%)	0.643

*Statistically significant.

## Discussion

The overall vaccine acceptance rate was 48.2% higher than the multinational study in which the acceptance rate was 26.7% (13). We found a higher vaccine acceptance rate in a British study that showed an acceptance rate of 71.7% (14). These differences could attribute to using different evaluating tools and implanting awareness programs in higher-income countries. We found that gender is insignificantly associated with vaccine acceptance, which disagrees with a study conducted in France, where the acceptance of vaccination among males was significantly higher than among females (18). Although the Sudanese Ministry of Health priority older age groups for taking COVID-19 vaccines, the prevalence of vaccine acceptance among the elderly is still low. Age is insignificantly associated with the decision to vaccinate. Grech et al. (19) found a higher rate of COVID-19 vaccine uptake in the older age group, as they are the more vulnerable group, therefore more likely to accept the vaccine.

Our results revealed that about two-thirds of the healthcare workers (64.3%) accepted vaccination against COVID-19 (*p* = 0.011) disagreeing with the Congo study (20), which found that the acceptance of COVID-19 vaccination among healthcare workers was only 28%. It also disagreed with the research conducted in the USA (15), with 36% acceptance and 56% hesitancy. Meanwhile, the France study reported a higher acceptance rate (18), where 77.6% of participants probably agreed to get vaccinated, which agreed with Barry et al. (16) whose study was to assess the COVID-19 vaccine confidence in a MERS-CoV experienced nation and found that two-thirds of HCWs expressed willingness to receive a potential COVID-19 vaccine.

49.0% of medical students show a willingness to get the COVID-19 vaccine, a higher acceptance rate stated by Barello et al. (17) as they found that students in the Italian universities students' intended to get the COVID-19 vaccine is 86.1%, and on the other side, 13.9% of them reported no willingness. The higher estimated willingness to vaccinate among medical students could attribute to higher literacy on health-related issues (21).

In this study, 36.5, 44.2, and 47.1% of the HCWs, medical students, and the general public, respectively, believed that COVID-19 is a synthetically developed virus (*p* < 0.001), which indicates that conspiracy beliefs and misleading information that are on social media platforms which are not a preferred knowledge source due to the public misinformation (conspiracy theory). The World Health Organization warned that the world is fighting another kind of epidemic called an info-demic regarding the overabundance of information (22, 23).

We found that 46.4, 27.7, and 37.7% (*p* = 0.041) of the HCWs, medical students, and the general public, respectively, believe that the pandemic is exaggerated to benefit pharmaceutical companies. An American study showed a nearly similar high rate of HCWs that did not trust information about COVID-19 and its severity, also by the regulatory authorities and pharmaceutical companies for vaccine development and safety (15). Scientific articles and health organizations are the most common sources of information for HCWs and medical students with rates of 50.0 and 38.8%, respectively (*p* = 0.041), which agree with the findings of a Libyan study (24).

As a reflection of participants' expected attitude toward COVID-19 vaccines, we asked the participants about the best way to deal with the vaccine in Sudan, only 31.9% of healthcare workers believe that vaccination should be optional for the whole population which is lower than 56.7% of the multinational study in response to the same question (13).

In the current study, the participants showed a high level of concern for COVID-19 vaccine side effects, which stops against high rates of vaccine acceptance, although we found that more than half of medical students (51%) are afraid of unknown side effects of the COVID-19 vaccine (*p* = 0.003), this rate is slightly lower than the rate of an Egyptian study that showed a rate of 56.3% (25). Evidence suggests the importance of concentrating on building trust in COVID-19 vaccines through using trusted messengers to navigate the COVID-19 information paradigm and confidence-building in vaccines through transparency and expectation management.

Although the representation of participants from different states in Sudan and the diversity of gender and occupations represent the strength of this study, using an online survey might impact the generalizability of the study, especially among the older age group trying to fix this we used a printed form of the survey but most of the elder age group showed non-compliance. Moreover, healthcare workers are underrepresented.

## Conclusion

Sudanese have an inadequate degree of knowledge and awareness about COVID-19, and public health authorities and the government in Sudan have a heavy mission for implementing successful vaccination programs with high coverage. We recommend implanting programs to raise awareness about COVID-19 Vaccines. These programs can include education on the danger of COVID-19 and how vaccines can protect from it or reduce its symptoms. Moreover, there is a need for more studies focusing on social factors affecting vaccine acceptance and the effect of awareness-raising campaigns on vaccine acceptance, and exploration of the link between differences in vaccine acceptance among different occupations.

## Data availability statement

The raw data supporting the conclusions of this article will be made available by the authors, without undue reservation.

## Ethics statement

The studies involving human participants were reviewed and approved by the research Ethics Committee at Faculty of Medicine, Ain Shams University. Written informed consent to participate in this study was provided by the participants' legal guardian/next of kin.

## Author contributions

HM and AA edited the original draft. All authors contributed to data collection, writing the original draft, and approved the final version for publication.

## Conflict of interest

The authors declare that the research was conducted in the absence of any commercial or financial relationships that could be construed as a potential conflict of interest.

## Publisher's note

All claims expressed in this article are solely those of the authors and do not necessarily represent those of their affiliated organizations, or those of the publisher, the editors and the reviewers. Any product that may be evaluated in this article, or claim that may be made by its manufacturer, is not guaranteed or endorsed by the publisher.
